# The Effects of MgO and Al_2_O_3_ Content in Sinter on the Softening–Melting Properties of Mixed Ferrous Burden

**DOI:** 10.3390/ma16155490

**Published:** 2023-08-06

**Authors:** Zhexi Li, Tingle Li, Changyu Sun, Songtao Yang, Qi Wang

**Affiliations:** School of Materials and Metallurgy, University of Science and Technology, Anshan 114051, China; lnkjdxlizhexi@126.com (Z.L.); sunchangyu1979@163.com (C.S.); yangsongtao1984@163.com (S.Y.)

**Keywords:** MgO, Al_2_O_3_, high-basicity sinter, mixed ferrous burden, softening–melting properties

## Abstract

The softening–melting properties of mixed ferrous burden made from high-basicity sinter with increased MgO and Al_2_O_3_ content and acid pellets was investigated for optimization. The influences of MgO and Al_2_O_3_ are discussed with the aid of phase analysis. The results showed that, with decreasing MgO mass%/Al_2_O_3_ mass% in mixed burden, all the softening–melting characteristic temperatures decreased, which can be attributed to the low melting temperature and viscosity of the slag caused by MgO and Al_2_O_3_. The permeability of the melting zone deteriorated again when MgO mass%/Al_2_O_3_ mass% decreased to a certain content. The softening interval widened slightly at first and then narrowed, while the melting interval first increased slightly and then increased greatly later. It can be deduced that the softening properties were improved, but the melting properties were worsened. Under comprehensive consideration of its softening–melting properties, permeability, iron ore reduction and the thermal state of the blast furnace hearth, the optimal softening–melting properties of a mixed ferrous burden with MgO mass%/Al_2_O_3_ mass% of 0.82 is optimal.

## 1. Introduction

The blast furnace (BF)–oxygen steelmaking route which uses iron ores as the raw ferrous materials is the primary source for worldwide steel production. The BF is the biggest counter-current reactor involving downward iron ores (sinter, pellets and lump ore) and upward gas flow for producing pig iron [1]. Normally, the ferrous burden and coke are charged into the BF separately, giving the furnace stack a layered structure. The ore layer undergoes a heating and reduction process in the BF. On reaching the lower part of the BF at much higher temperatures, they soften and melt, forming the region known as the cohesive zone. The softening and melting (SM) properties of ferrous burden have a signification effect on the shape, position and thickness of the cohesive zone [2,3], determining the gas utilization and heat transfer, which vitally influences BF operational performance and productivity [4].

A sinter with high basicity has good reduction and SM properties while its Fe content is low. A high Fe content and a low gangue content are favorable for acid pellets. However, it has inferior reduction and SM properties due to low-melting fayalitic slag [5]. The pelletizing process is much cleaner than the sintering process, while the former has low yields [6]. Sinter and pellets have become the popular ferrous materials for BF ironmaking [7]. It is a feasible direction to use mixed ferrous burden made with high-basicity sinter and acid pellets for green metallurgy due to their complementary advantages [8].

Recently, because of the increasing scarcity of good-quality iron ores, high Al_2_O_3_ iron ores of low grade and low cost have been utilized to make high-basicity sinter, pursuing economic benefit [9,10]. Increased Al_2_O_3_ content in BF ferrous burden leads to high viscosity [11,12,13,14] and bad desulfurization ability [15] of the slag and low permeability [16] in the cohesive zone. A popular countermeasure is adding MgO into sinter [17]. So, both the MgO and Al_2_O_3_ content in sinter increase and the MgO mass%/Al_2_O_3_ mass% changes, which are bound to influence the SM properties of high-basicity sinter and its mixed ferrous burden with acid pellets [18], consequently attracting our attention.

The effect of MgO or Al_2_O_3_ content on the SM properties of sinter and pellets has been reported in many studies in the literature. The works of Guo et al. [19,20] demonstrated that the softening temperatures and the maximum pressure drop increased, and the melting temperature interval became wide with the increasing MgO content in sinter, because the MgO existed as a wüstite solid solution when sinter softened, and then on melting it brought a high-melting point slag. Li et al. [21] conducted a study of the effect of MgO content on the SM performance of high-gangue sinter and partially supported Guo’s viewpoint. They believed that MgO content did not always improve the SM performance of sinter but it had a certain content limit. Wang et al. [22] proposed that high Al_2_O_3_ content sinter was associated with a thicker melting–dripping zone, leading to the deterioration of sinter bed permeability. Our previous work [17] on the effects of MgO and Al_2_O_3_ on the SM properties of high-basicity sinter found that MgO and Al_2_O_3_ led to increased and decreased SM characteristic temperatures, respectively. The influence of MgO on SM properties was insignificant compared with Al_2_O_3_, which was attributed to the fact that MgO existed in wüstite as an FeO–MgO solid solution and Al_2_O_3_ was distributed in slag. In the case of pellets, Iljana et al. [23] carried out experiments on metallurgical properties with different-sized pellets. They observed that the higher proportions of MgO in the silicate slag and in the wüstite resulted in increased softening temperatures. They also verified that increasing the MgO content can improve the softening properties of pellets via computational thermodynamics [24] and proposed the separation of Al_2_O_3_ from iron ore concentrate to avoid deteriorating the softening properties. Chen et al. [25] reviewed the effect of MgO on the metallurgical properties of pellets, and pointed out that the softening properties were improved obviously, but the melting properties were not improved with increasing MgO content.

However, research on the SM properties of mixed burden composed of sinter and pellet is relatively limited compared to single ore species [26,27], especially regarding the comprehensive effect of MgO and Al_2_O_3_ under the current conditions that both MgO and Al_2_O_3_ in high-basicity sinter are increased. There are still some difficulties in adjusting the MgO and Al_2_O_3_ content in sinter for the purpose of optimizing the SM properties of mixed ferrous burden. Therefore, the SM properties of mixed ferrous burden made from high-basicity sinter with increased MgO and Al_2_O_3_ content and acid pellets were investigated in this study. The influence mechanisms of MgO and Al_2_O_3_ were analyzed with the aid of phase analysis. We hope that the results will be useful to the establishment of a picture about the SM behavior of mixed burden, and its effect on BF operations.

## 2. Materials and Methods

### 2.1. Materials

Three kinds of high-basicity sinters and one kind of acid pellet, derived from actual ironmaking plants, were selected as experimental materials. The chemical compositions are listed in Table 1. It shows that there was only a little variation in the degree of the TFe content, FeO content and binary basicity (C/S) in the sinter samples. The changes in MgO and Al_2_O_3_ content in the sinters were more interesting. From S_1_ to S_3_, the MgO content increased from 1.70 to 3.16 mass% with increasing Al_2_O_3_ content (from 1.14 to 4.03 mass%), while the MgO mass%/Al_2_O_3_ mass% (M/A) decreased from 1.49 to 0.78.

The experimental samples were three kinds of mixed burdens. All the mixed burdens consisted of 70 mass% sinter and 30 mass% pellets, which is a general burden structure in China. The chemical compositions of mixed burdens are shown in Table 2. From S_1_P to S_3_P, MgO and Al_2_O_3_ content increased continually, from 1.33 to 2.36 mass% and from 1.23 to 3.25 mass %, respectively. The M/A kept a decreasing trend from 1.08 to 0.73.

### 2.2. Experimental Procedure

**Figure 1:**
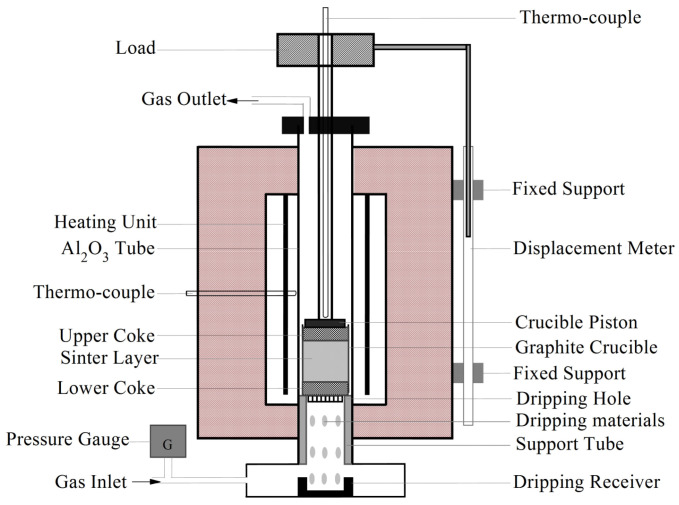
The experimental equipment of SM test.

The experimental equipment of the SM test is shown in Figure 1. A packed bed comprising mixed burden (200 g) packed in between two coke layers (20 mm each) was heated with a load of 0.5 kg·cm^−2^ in a graphite crucible. The particle sizes of iron ores and cokes were 10~12.5 mm and 6~8 mm, respectively. All the materials were dried at 105 °C for 24 h before testing. The dripping holes in the bottom of the crucible allowed the gas flow to pass through the burden and the molten iron/slag to drip. The mixed burden was heated at 10 °C·min^−1^ below 950 °C, and kept at 950 °C for 1 h, and then heated at 5 °C·min^−1^ over 950 °C. The gas flow was changed from N_2_ (5 L·min^−1^) to a reducing gas composed of CO and N_2_ with the ratio 30/70 (12 L·min^−1^) until the sample temperature reached 400 °C. A more detailed description of the test procedure was given in our previous work [28].

In the SM process, the ferrous burden bed deformed (softened and melted) and then hindered the gas flow. During the whole experimental process, several important parameters such as the sample temperature, shrinkage ratio (Δh) and pressure drop (ΔP) were continuously recorded by the thermocouple, the displacement meter and the pressure gauge, respectively. When the test was finished, the dripped substance and residual materials in the graphite crucibles were collected for further analysis.

## 3. Results and Discussion

**Figure 2:**
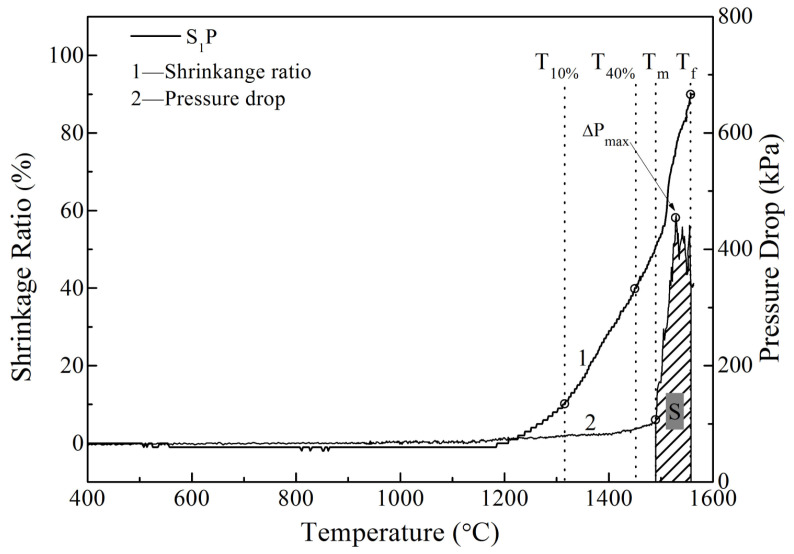
A typical set of SM curves of the mixed burden.

A typical set of SM curves of the mixed burden is shown in Figure 2, which includes the shrinkage ratio (Δh) and pressure drop (ΔP) changes against the sample temperature. To represent and compare the SM properties of these samples, some indexes were adopted. As evident from Figure 2, when Δh reached 10% and 40%, the sample temperatures T_10%_ and T_40%_ were defined as softening start temperature and softening end temperature, respectively. The melting start temperature T_m_ was the temperature that ΔP began to steeply increase; and T_f_ was the melting end temperature at which Δh tended to be unchanged. The softening temperature interval (T_40%_–T_10%_) represented the softening zone while the melting temperature interval (T_f_–T_m_) represented the melting zone. Because ΔP was at the low level in the softening range, the softening zone was expected to be wide while the melting zone should be narrow, indicating good SM properties of the burden. During the melting temperature interval, when the first dripping phenomenon was observed, the sample temperature was recorded as the dripping temperature T_d_. ΔP_max_ was the maximum value of the pressure drop curve. S was defined as the permeability index of the melting zone and supposed to be the integral value of the pressure drop function over the melting zone (from T_m_ to T_f_), seeing the shaded area in Figure 2. The higher the value of S, the worse the permeability of the mixed burden in the melting zone.

### 3.1. The Effects of MgO and Al_2_O_3_ on Softening Properties

**Figure 3:**
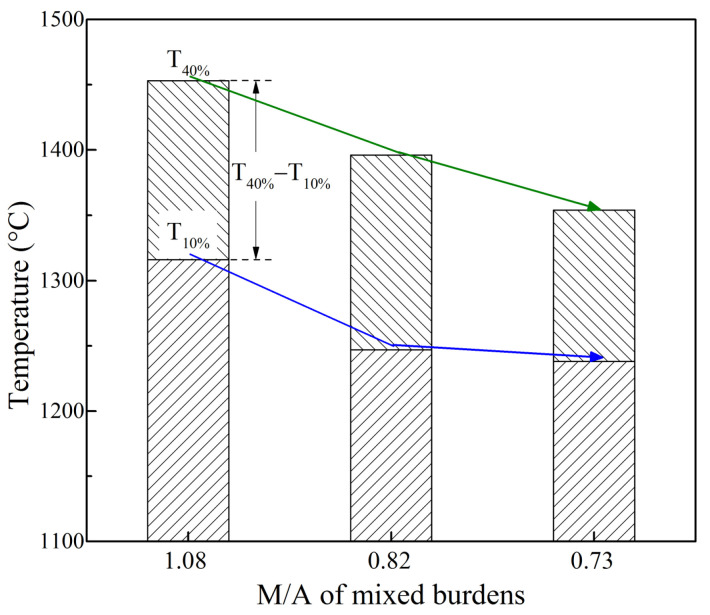
Effects of MgO and Al_2_O_3_ content in sinter on softening properties of mixed burdens.

Figure 3 shows the effects of MgO and Al_2_O_3_ content in high-basicity sinter on the softening characteristic temperatures of mixed burdens. As the MgO and Al_2_O_3_ content increased and the M/A decreased, the softening start temperature T_10%_ and the softening end temperature T_40%_ showed decreasing trends from 1316 °C to 1238 °C and from 1453 °C to 1354 °C, respectively. The decrease extent of T_10%_ from S_1_P to S_2_P was more significant than that observed from S_2_P to S_3_P. The softening interval (T_40%_–T_10%_) widened slightly from 137 °C to 149 °C at first, and then narrowed down to 116 °C. In terms of the position and thickness of the cohesive zone in BF, the upper edge of the cohesive zone and the corresponding T_10%_ moved upward. The experimental softening results showed that the M/A of mixed burden had a certain content limit. The possible thinning of the thickness of the softening zone was achieved, which indicated that the softening properties of mixed ferrous burden were improved when adjusting the M/A.

The softening characteristic temperatures of mixed burdens tended towards those of its corresponding high-basicity sinter and could be explained as followed. First, according to previous research on sinter mineralogy [17,29], increasing the MgO and Al_2_O_3_ content facilitated the formation of magnesioferrite and dampened hematite obviously, whereas the amounts of calcium ferrite almost kept constant. The sinter containing higher amounts of MgO and Al_2_O_3_ had a relatively lower reducibility. This fact resulted in earlier deformation, leading the lower T_10%_ and T_40%_. Second, the increase in Al_2_O_3_ content in sinter favored the formation of an Al_2_O_3_-rich primary slag phase with low melting temperature, such as the eutectic of 2CaO·SiO_2_-2CaO·Al_2_O_3_·SiO_2_-FeO [22]. It has been suggested that MgO has a positive influence on softening temperatures [30]. However, the effect of Al_2_O_3_ on softening was likely to be more dominant than MgO [17]. Thus, the softening temperatures were expected to decrease. It is indispensable to optimize the softening properties of mixed burden, but only when considering both the reduction property and gas permeability. In the softening process of the samples, its pressure drop was relatively low before intensely climbing. The wide softening zone benefited indirect reduction and decreasing coke rate. Therefore, it is believed that the softening property of S_2_P was the best where the certain M/A of the mixed burden was 0.82.

### 3.2. The Effects of MgO and Al_2_O_3_ on Melting Properties

**Figure 4:**
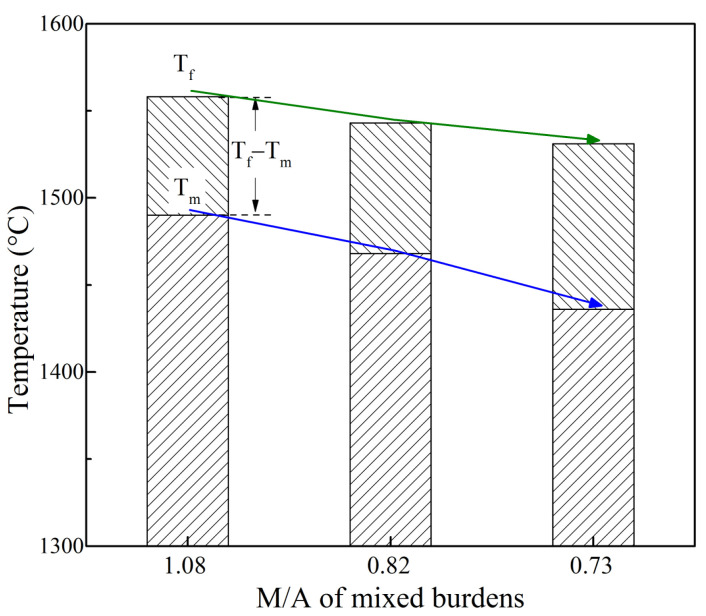
Effects of MgO and Al_2_O_3_ content in sinter on melting properties of mixed burdens.

The effects of MgO and Al_2_O_3_ content in sinter on the melting characteristic temperatures of mixed burdens are shown in Figure 4. With the increase in MgO and Al_2_O_3_ content in sinter, the melting start temperature T_m_ and the melting end temperature T_f_ decreased from 1490 °C to 1436 °C and from 1558 °C to 1531 °C, respectively. The melting interval (T_f_–T_m_) first increased slightly, and then increased greatly later. According to the experimental melting results, the lower edge of the cohesive zone and the corresponding T_f_ moved upward and the thickness of melting zone and corresponding T_f_–T_m_ increased. It should be noted that once the M/A of the mixed burden was less than 0.82, the melting properties were worsened significantly, which was not the expectation. The dripping temperature T_d_ of S_1_P, S_2_P and S_3_P was 1534 °C, 1506 °C and 1481 °C, respectively. The T_d_ is an important factor which determines physical heat in the BF hearth. If the T_d_ is too low, the heat carried by the molten iron cannot allow the blast furnace hearth to achieve good performance in desulfurization and slag tapping, which is detrimental to production. From the T_d_, it is proposed that the M/A of mixed burden cannot be less than 0.82.

**Figure 5:**
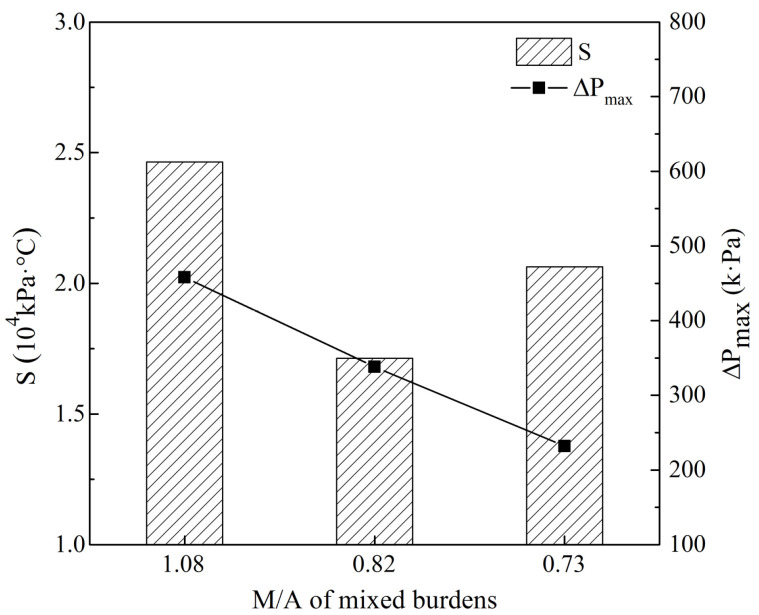
Effects of MgO and Al_2_O_3_ content in sinter on the permeability of melting zone.

Figure 5 shows the effects of MgO and Al_2_O_3_ content in sinter on the permeability of the melting zone. With the increase in MgO and Al_2_O_3_ content, ΔP_max_ decreased from 458 kPa to 232 kPa while the S value decreased at first and then increased, indicating that the permeability of the mixed burdens first became better and then deteriorated. From the width and permeability of the melting zone, the mixed burden with an M/A of 0.82 had the best melting property. Therefore, it is suggested that the proper M/A of the mixed burden made from high-basicity sinter and acid pellets should be about 0.82 when both the MgO and Al_2_O_3_ content are increased in sinter.

**Figure 6:**
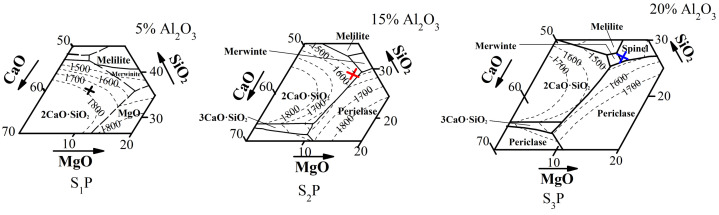
The phase diagrams of CaO–SiO_2_–MgO–Al_2_O_3_ system.

T_m_ and T_d_ were related to the molten slag properties, such as liquidus temperature and viscosity. The slag was mainly derived from the gangue components in mixed burden and can be theoretically estimated with CaO–SiO_2_–MgO–Al_2_O_3_ system phase diagrams [31]. Figure 6 shows the phase diagrams of a CaO–SiO_2_–MgO–Al_2_O_3_ system, in which the fractions of Al_2_O_3_ are 5 mass%, 15 mass% and 20 mass%, respectively. From S_1_P to S_3_P, the liquidus temperature of the molten slag is obviously decreased. An aforementioned fact is that the low reducibility of sinter with high MgO and Al_2_O_3_ content can result in high FeO content remaining in the molten slag after mixed burden melting. FeO is a typical substance which can decrease the melting temperature of slag. In addition, it has been reported that blast furnace slag viscosity decreases with increasing FeO content [32]. Therefore, T_m_ and T_d_ showed decreased trends.

### 3.3. Roles of MgO and Al_2_O_3_ during Melting

**Figure 7:**
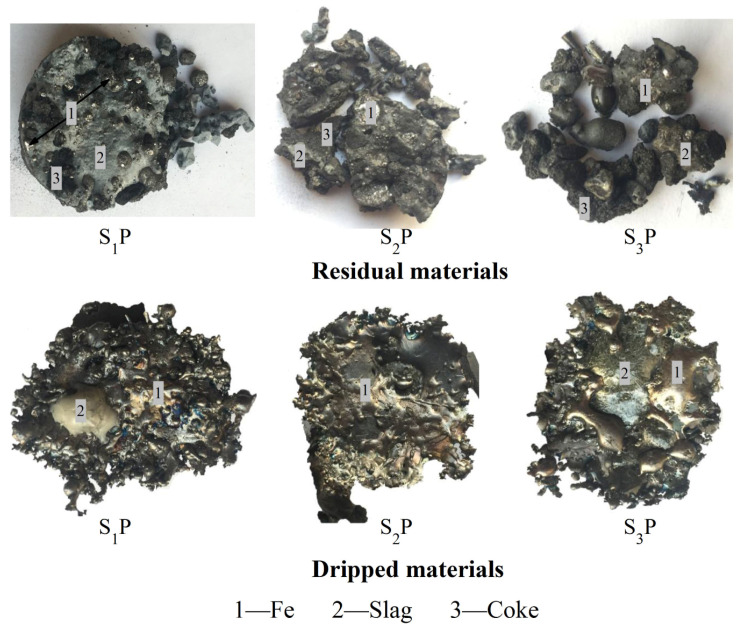
The photos of dripped materials and residual materials.

Figure 7 shows photos of the residual materials and dripped materials after the experiments. The residual materials consisted of large amounts of slag phase and a relatively smaller amount of iron. In contrast, the dripped materials were mainly composed of iron and relatively less slag.

**Figure 8:**
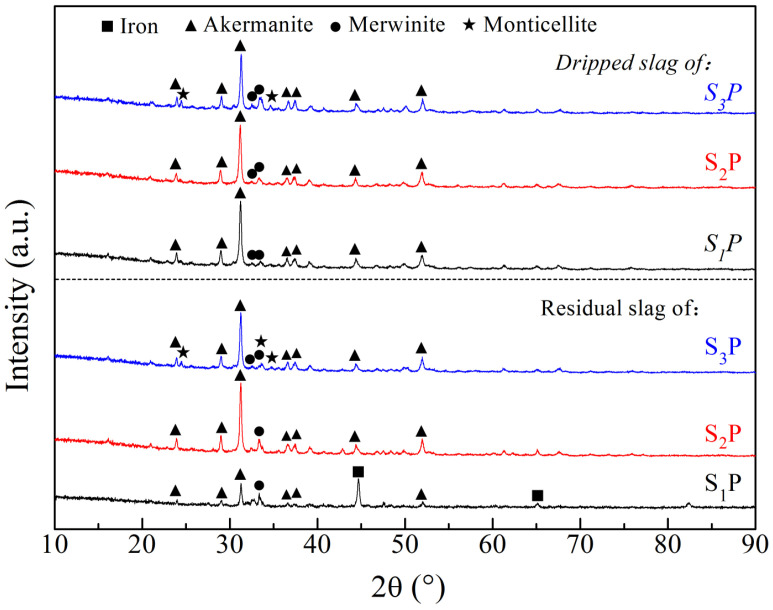
The X-ray diffraction analysis of the residual slags and dripped slags.

To illustrate the roles of MgO and Al_2_O_3_ during melting, X-ray diffraction pattens of the residual slags and dripped slags are shown in Figure 8. For the residual slags and dripped slags of S_1_P and S_2_P, the main minerals were melilite (Ca_2_(Al,Mg)[(Si,Al)SiO_7_]) and merwinite (3CaO·MgO·2SiO_2_). In contrast, the residual slag and dripped slag of S_3_P contained an additional component, monticellite (CaO·MgO·SiO_2_). The melting point of monticellite is 1390 °C, which is lower than those of melilite and merwinite. It is suggested that a higher MgO and Al_2_O_3_ content in sinter results in the generation of relatively low-melting point components in the slag of mixed burden. It can be deduced that the mixed burden containing a high MgO and Al_2_O_3_ content with a low M/A can drip easily, resulting in the low T_d_ and low ΔP_max_. It is probable that the widest melting temperature interval of S_3_P where the M/A is less than 0.82 brings about the higher S value and worse permeability. Thus, under comprehensive consideration of the SM properties and the roles of MgO and Al_2_O_3_, the M/A of mixed burden made with high-basicity sinter and pellets should be about 0.82.

## 4. Conclusions

The softening–melting properties of mixed ferrous burden made from high-basicity sinter with increased MgO and Al_2_O_3_ content and acid pellets and the roles of MgO and Al_2_O_3_ during melting were investigated. Several conclusions were obtained.

(1) With decreasing MgO mass%/Al_2_O_3_ mass% in mixed burden, the softening–melting characteristic temperatures of mixed burdens tended to decrease. The softening interval widened slightly at first, and then narrowed, while the melting interval first increased slightly, and then increased greatly. The permeability of the melting zone deteriorated once the MgO mass%/Al_2_O_3_ mass% was less than a certain content.
(2) The low melting temperature and viscosity of the slag of mixed ferrous burden with a low MgO mass%/Al_2_O_3_ mass% was the main reason for the reduction in softening–melting characteristics.
(3) On the whole, with decreasing MgO mass%/Al_2_O_3_ mass% in mixed burden, the softening properties were improved whereas the melting properties were worsened. Considering the iron ore reduction and thermal state of blast furnace hearth, mixed burden had the optimal softening–melting properties at an MgO mass%/Al_2_O_3_ mass% of 0.82.

## Figures and Tables

**Table 1:** The chemical compositions of high-basicity sinters and pellets (mass%).

Materials	TFe ^1^	FeO	SiO_2_	CaO	MgO	Al_2_O_3_	M/A ^2^	C/S ^3^
S_1_	54.39	9.54	6.63	13.15	1.70	1.14	1.49	1.98
S_2_	54.70	9.40	5.53	10.26	2.84	3.08	0.92	1.86
S_3_	52.30	9.00	6.16	11.31	3.16	4.03	0.78	1.84
P	63.80	0.18	3.38	0.34	0.48	1.43	0.69	0.10

^1^ TFe means the total Fe content in mass%; ^2,3^ M/A and C/S mean CaO mass%/SiO_2_ mass% and MgO mass%/Al_2_O_3_ mass%, respectively.

**Table 2:** The chemical compositions of mixed burdens (mass%).

Mixed Burdens	TFe ^1^	FeO	SiO_2_	CaO	MgO	Al_2_O_3_	M/A ^2^
S_1_P	57.21	6.73	5.66	9.31	1.33	1.23	1.08
S_2_P	57.43	6.63	4.89	7.28	2.13	2.59	0.82
S_3_P	55.75	6.35	5.33	8.02	2.36	3.25	0.73

^1^ TFe means the total Fe content in mass%; ^2^ M/A means MgO mass%/Al_2_O_3_ mass%.

## Data Availability

The data presented in this study are available on request from the corresponding author.
